# Descriptive Analysis of the First 12-Month Activity of Docadom, a Private Physician-Led Urgent In-Home Consultation Service

**DOI:** 10.3390/jcm15156089

**Published:** 2026-08-05

**Authors:** Alexis Bikfalvi, Lorenzo Berri, Nicole Albrecht, Nicolas Calzoni, Gabriele Lecca, Vilte Sauliunaite, Christine Carnal, Eric Albrecht

**Affiliations:** 1 Docadom, Avenue de Béthusy 29, 1012 Lausanne, Switzerland; ab@docadom.ch (A.B.); lberri@docadom.ch (L.B.); nalbrecht@docadom.ch (N.A.); ncalzoni@docadom.ch (N.C.); glecca@docadom.ch (G.L.); vsauliunaite@docadom.ch (V.S.); ccarnal@docadom.ch (C.C.); 2 University of Lausanne, 1015 Lausanne, Switzerland

**Keywords:** emergency medicine, in-home consultation, urban health services

## Abstract

**Background:** Emergency services in urban areas are overcrowded due to a rise in mild-to-moderate acuity consultations, and physician-led home visits are less common. In response, a home-based urgent care model in an urban setting (Docadom) was developed. Consultation requests via a dedicated mobile application or phone call are triaged by a trained emergency nurse. Physicians travel to the patient via an electric cargo bike that has storage for medical equipment. During home visits, physicians perform testing/diagnosis and advanced procedures (e.g., intravenous medication delivery) and prescribe treatment (if needed). This descriptive analysis reports activities during the first 12 months of Docadom. **Methods:** Data from all patients seen during the first 12 months of Docadom activity (1 May 2023–30 April 2024) were analysed. Outcomes analysed included: age, sex, diagnosis, and whether the patient was referred to the hospital. Patient satisfaction (from 1 [worst] to 5 [best]) is also reported. Each outcome was analysed in two subgroups based on patient age (<75 or ≥75 years) because the Swiss health insurance system distinguishes between these two age groups, using Wilcoxon or chi-square tests as appropriate. **Results:** A total of 1736 patients requested a consultation (66% female; median age 70 years [interquartile range 36, 84]; 43% aged ≥75 years). The five most frequent clinical presentations were: ear, nose and throat infection, epistaxis and bronchitis (25.7%); soft tissue trauma (7.8%); acute cervical or lower back pain syndrome (5.6%); gastroenteritis and dehydration (5.0%); and cancer and general decline in the elderly (4.6%). Only 4.7% of the patients were referred to the hospital. The mean satisfaction score was 4.97 (response rate: 207/422 patients [49%] who requested a consultation via the app). **Conclusions:** These data highlight the feasibility and acceptability of a home-based urgent care model in an urban setting. The service effectively addressed a wide range of acute conditions, especially in elderly patients. The low hospital referral rate and high patient satisfaction are encouraging, but as a single-arm descriptive analysis without post-consultation outcome data or a comparator group, this study cannot establish clinical safety or an effect on emergency department use; these questions require a future comparative study.

## 1. Introduction

In recent years, emergency services in urban areas have faced increasing pressure due to a rise in mild-to-moderate acuity consultations, often resulting in overcrowded emergency departments, prolonged waiting times, and unsustainable healthcare costs [1,2,3]. With 850,000 inhabitants in the Canton of Vaud, Switzerland, there are over 220,000 urgent consultations each year, with an annual growth rate of 5%, much of which is attributable to ambulatory presentations [4]. The situation is particularly critical in Lausanne (150,000 inhabitants), where emergency departments are chronically overloaded, and patients often wait between three and six hours for non-critical care [5].

Simultaneously, physician-led home visits—once a cornerstone of urgent outpatient care—have sharply declined due to urban congestion and increased complexity in therapeutic management [6]. As a result, there remains a critical gap in the continuum of urgent care services, especially for the elderly or individuals with reduced mobility. Emerging portable diagnostic technologies have created new opportunities to reintroduce a robust and feasible physician-led urgent in-home consultation service.

Launched on 1 May 2023 as a private initiative, Docadom is a health project that was developed in response to the above-mentioned systemic challenges. The aim of Docadom is to deliver high-quality, technology-enabled, urgent medical care directly to the patient at home. Requests are initiated via a user-friendly mobile application or call centre and triaged by trained emergency nurses. In-home consultations are assigned to experienced physicians who travel by electric cargo bikes that are equipped with portable diagnostic technology, including a 12-lead electrocardiogram (ECG), point-of-care testing (POCT), and point-of-care ultrasound (POCUS).

The service covers a wide range of acute and urgent conditions, from fever, minor trauma and dermatologic concerns to more severe conditions when patients refuse to go to an emergency department (e.g., suspicion of acute coronary syndrome, pulmonary embolism or sepsis). The model integrates clinical care with digital documentation, real-time coordination with pharmacies, and direct communication with a patient’s primary care physician. The goals of the Docadom service are to reduce avoidable emergency department visits, improve triage efficiency, and contribute to a more sustainable patient-centred urgent care ecosystem.

This analysis describes the structure and early operational outcomes of the Docadom service during the first 12 months of operation.

## 2. Materials and Methods

### 2.1. Study Design

Data from all patients treated during the first 12 months of Docadom operation (1 May 2023 to 30 April 2024) were retrospectively analysed. The study was approved by the local ethics committee (Commission cantonale d’éthique de la recherche sur l’être humain, CER-VD) and was conducted in accordance with the Declaration of Helsinki. This study is reported in accordance with the Strengthening the Reporting of Observational Studies in Epidemiology (STROBE) guideline and its RECORD extension for studies conducted using routinely collected health data; the completed checklist is provided as Supplementary File S1.

### 2.2. Service Details

**Figure 1:**
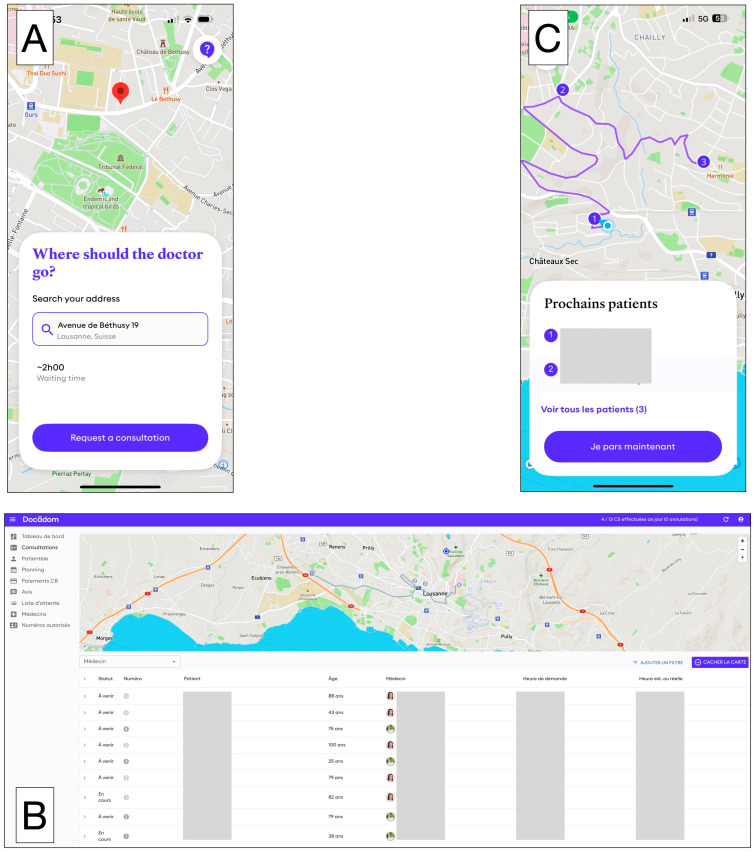
Interfaces for the patient mobile application (**A**), the web application (**B**), and the physician mobile application (**C**). All applications were specifically developed by Docadom for the purpose of delivering an efficient physician-led urgent in-home consultation service. The patient mobile application is available in French and English.

Docadom provides care to adult patients (age > 16 years) between 8:30 am and 8:30 pm, seven days a week. Consultations can be requested by the patient, a family member, nursing homes, community nursing centres, or a primary care physician. The consultation can be requested through a mobile application in French or English (Docadom, iOS/Android, Version 1.0, 2023, Lausanne, Switzerland) or a call centre (institutional lines). A trained emergency nurse undertakes a triage process by reviewing incoming requests and screening for emergencies. In cases of life-threatening conditions, patients are advised to contact a staffed medical ambulance service. Once a consultation has been accepted, a chart is created in a specific web application that communicates with an electronic health record system. Patients are queued and assigned to the nearest available physician based on geolocation, case urgency, and estimated consultation time. Physicians have access to their list of patients through another specific mobile application. The patient mobile application, the web application, and the physician mobile application were specifically developed by Docadom (Lausanne, Switzerland) (Figure 1).

Physicians working with Docadom are board-certified in general internal medicine, with a certificate in emergency medicine. All physicians operate as independent contractors and use Docadom-provided equipment. They are dispatched on electric cargo bicycles, allowing for efficient and environmentally responsible travel within the city. There are 2–3 parallel physician lines per day.

Each physician is equipped with a standardised medical kit that includes the following (Table 1): a stethoscope, a pulse oximeter, and a sphygmomanometer; quick biological tests (COVID-19, influenza A/B, respiratory syncytial virus, streptococcus, spot urine sample, and pregnancy); portable 12-lead electrocardiography; an ultrasound probe (smartphone-connected); a POCT device for basic labs; a suture kit; a laptop with encrypted access to electronic patient records; and medications that can be given to the patient to cover the first 24 h of treatment. When necessary, blood samples and biological cultures are drawn and collected by a courier from a private laboratory, with results available within two hours via an electronic interface. Referral to a radiology centre is possible, if needed.

All medical encounters are documented in real time using an electronic patient record that is hosted on secure Swiss-based servers. Post-consultation reports are automatically shared with the patient and their primary care physician. Prescriptions are sent electronically to the patient’s local pharmacy, with medication delivery within 24 h if needed. Billing follows the Swiss third-party payer system: an electronic invoice is sent directly to the patient’s health insurance, with co-payments billed separately.

**Figure 2:**
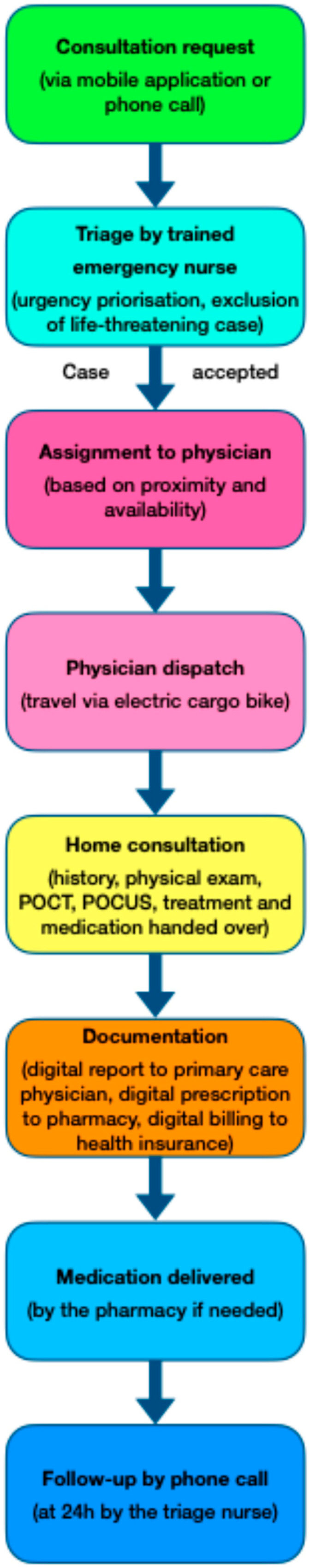
Docadom patient workflow. POCT, point-of-care testing; POCUS, point-of-care ultrasound.

After each consultation requested via the application, patients receive a quick satisfaction survey that is completed via the mobile app and asks them to assess the physician on a scale from 1 (lowest score) to 5 (best score). At 24 h, a follow-up phone call is performed by the triage emergency nurse. Figure 2 depicts the Docadom patient workflow.

### 2.3. Data Collection

The data and outcomes analysed were: age; sex; person who requested the consultation (patient, relative, nurse, or primary care physician); mode of request (application or phone call); waiting time (from the consultation request to the doctor’s arrival at the patient’s home); travel time (from the doctor’s departure from one patient to arrival at the next patient’s home); consultation time (from arrival to departure); bedside tests performed (quick test, POCT, electrocardiography, and POCUS); laboratory tests; advanced procedures (intravenous infusion of medication, intravenous rehydration, wound stitches, abscess incision, and urinary bladder catheterisation, among others); referral to a radiological centre (ultrasound, computed tomography scan, magnetic resonance imaging, and X-ray); outcome of the consultation (health issue solved, follow-up by Docadom, follow-up by the primary care physician, or referral to a hospital); and diagnosis. The satisfaction score was also recorded and reported.

### 2.4. Statistical Analysis

Each outcome was analysed in two subgroups based on patient age (<75 or ≥75 years) because the Swiss health insurance system distinguishes between these two age groups. Continuous variables are reported as median values with interquartile ranges (IQRs), and categorical variables are reported as numbers and percentages. Continuous data were compared between groups using the Wilcoxon test. Categorical and dichotomous data were compared using the chi-square test. Statistical significance was defined as a two-sided *p*-value < 0.05. Statistical analysis was performed using Stata software (Stata version 18.0, StataCorp, College Station, TX, USA).

## 3. Results

In its first year, Docadom completed 1736 urgent home consultations in the Lausanne metropolitan area. The average number of consultations increased steadily from one per day in the first month to nine per day after 1 year, reflecting both organic growth and increasing public awareness of the service.

The patients treated were predominantly female (66%); the median [IQR] age was 70 [36, 84] years; and 43% of patients were aged ≥75 years (Table 2). Patients aged ≥75 versus <75 years more often had their consultation requested by a nurse or a primary care physician (*p* < 0.001), had a longer consultation time (*p* < 0.001), and needed more follow-up or referral to a hospital (*p* < 0.001) (Table 2). Advanced procedures, such as intravenous therapy, urinary catheterisation or wound closure, were performed in 6.2% of consultations, with no significant difference between age subgroups.

Table 3 shows the diagnoses after completion of consultations. The most frequent clinical presentations were ear, nose and throat infections, epistaxis, and bronchitis, accounting for 25.7% of cases. These conditions were significantly more prevalent in patients aged <75 versus ≥75 years (35.9% vs. 12.4%; *p* < 0.001). Soft tissue trauma was the second most common condition overall (7.8%) and was significantly more common in the older versus younger age group (10.9% vs. 5.5%; *p* < 0.001). The rate of consultations for gastroenteritis and dehydration was similar in those aged <75 or ≥75 years (4.9% and 5.1%, respectively). Consultations for congestive heart failure, cancer and general decline were all significantly more common in older versus younger patients, while low back pain occurred more frequently in the younger subgroup.

The satisfaction survey was completed by 207 of the 422 patients (49%) who requested a consultation via the app, with a mean rating of 4.97/5; specific feedback often highlighted comfort, convenience, and perceived physician professionalism.

## 4. Discussion

This descriptive study of 1736 consultations provides valuable insights into the initial operational outcomes of Docadom, a novel and innovative physician-led urgent in-home consultation service in the Lausanne metropolitan area, Switzerland. After the consultation, 80% of patients remained at home without need for follow-up, 15% remained at home but with follow-up care arranged, and 5% were referred to an emergency department for further treatment. This low referral rate suggests appropriate triage and reflects the capability of Docadom physicians to resolve a high percentage of urgent care cases at home. Notably, over 40% of the consultation requests were submitted by healthcare professionals (i.e., community nursing centre staff), highlighting the service’s integration into institutional care pathways. The post-consultation satisfaction score (mean 4.97/5) reflects an overwhelmingly positive patient experience.

The ability of Docadom physicians to perform a wide range of diagnostic and therapeutic interventions at home, including ECG, POCT, POCUS, sutures and intravenous hydration, demonstrates the clinical robustness of the in-home consultation model and suggests that a substantial proportion of urgent cases can be effectively managed outside traditional hospital settings. The integration of digital documentation, real-time coordination with pharmacies, and communication with primary care physicians further enhances the quality and continuity of care, addressing the increasing complexity in therapeutic management that contributed to the decline of traditional home visits.

The reasons for consultation varied significantly by age and indicate the types of acute and urgent conditions that can be effectively managed at home by Docadom physicians. While younger patients (age < 75 years) predominantly presented with upper respiratory infections and bronchitis, older patients were more likely to be experiencing exacerbations of chronic conditions such as heart failure, cancer, or generalised decline. The low rate of referral to hospital is consistent with studies highlighting the benefits of timely, at-home care in frail elderly populations, where hospitalisations are often preventable with early intervention [7]. In our population, 43% of people who had a consultation in the first year were aged ≥75 years, underscoring the relevance of the service for older adults, who are high-frequency users of emergency services according to national statistics [4,8]. It is possible that the addition of a call centre 6 months after the mobile application was developed could have contributed to better uptake amongst older adults who may not be as comfortable with digital technologies. Further analysis of the age of people requesting consultation over a longer time period is needed to confirm the current impression that a growing proportion of older adults are utilising the Docadom service. This information would have implications for tailoring the service’s resources and physician expertise to match the anticipated patient profile.

Comparing the first year of Docadom experience with existing literature, the findings support the idea that innovative models of home-based urgent care can effectively address the challenges of overcrowded emergency departments and declining traditional home visits [9,10]. While we do not directly compare outcomes of patients managed using the Docadom model with those managed in traditional emergency care pathways, the high number of consultations for conditions often seen in emergency departments suggests that the in-home care model could reduce the number of avoidable emergency department visits. Furthermore, the ability to manage certain conditions at home, including low-risk pulmonary embolism, albeit in a small number of cases, indicates the potential to extend the scope of home-based urgent care beyond minor ailments, potentially aligning with the concept of hospital-at-home programmes [11]. Previous studies have shown that hospital-at-home programmes can yield outcomes comparable to inpatient care, with improved patient comfort and reduced healthcare costs [12,13].

These findings should be interpreted within an evolving and, in some respects, limited evidence base on home-based urgent care and emergency department diversion. Systematic reviews of hospital-at-home care generally report outcomes comparable to inpatient management, with shorter lengths of stay and lower costs [14]. Safety data nonetheless remain incompletely reported—the updated Cochrane review of admission-avoidance hospital-at-home noted that few trials documented whether deaths occurred during the home-care episode [15]—and evidence that such services reduce emergency department use is inconsistent: a recent Swedish evaluation of a mobile home-care team for older adults illustrates both the promise of the model and the difficulty of demonstrating a clear reduction in unplanned emergency department visits [16]. As Docadom targets acute, single-episode presentations rather than hospital-level care, and our descriptive data include no post-consultation outcomes, we cannot determine whether the service diverts demand from emergency departments; this question warrants a future controlled comparison.

The use of electric cargo bikes represents a novel and environmentally responsible solution to urban mobility challenges. Unlike existing car-based home visit services that are hindered by traffic congestion, the Docadom model facilitates timely care delivery while also reducing the carbon footprint of medical practice—a growing concern within the Swiss and European healthcare systems [17]. The use of electric cargo bikes was not possible due to weather conditions on 6 days in 12 months, and on those days, Docadom physicians used a taxi service to attend call-outs. Nevertheless, the electric cargo bikes were a reliable mode of transportation, with a travel time of <22 min in most cases, mainly due to the ability to use bike lanes and dedicated traffic lights.

Limitations of this study include its descriptive, single-arm nature, focusing on the first 12 months of activity in a single urban setting. The findings and in-home urgent care service may not be directly generalisable to rural areas or different health systems without adaptation. Furthermore, satisfaction data were collected only from app-based users, which limited the sample size and may introduce selection bias. Indeed, patients who completed the survey may represent a selected subgroup with potentially higher satisfaction levels than non-responders. Importantly, no post-consultation clinical outcomes were captured: the 24 h nurse follow-up call was used for patient reassurance and continuity of care but was not recorded as structured outcome data, so re-presentation, unplanned admission, and adverse events could not be assessed. Consequently, clinical safety and the effect of the service on emergency department utilisation lay beyond the scope of this analysis. Our findings originate from a single, privately operated, physician-led service within the Swiss healthcare system, which may limit their generalisability to other healthcare settings.

Finally, further research, including comparative trials and analysis of the service’s impact on emergency department utilisation and healthcare costs, would be valuable to fully assess the role, efficiency and sustainability of this model.

In conclusion, this 12-month descriptive analysis confirmed the feasibility and acceptability of the Docadom physician-led urgent in-home consultation service in an urban setting. The service effectively addressed a wide range of acute conditions, particularly benefiting elderly patients. The integration of portable diagnostic technology and sustainable transport contributed to timely, patient-centred, and effective urgent care. The low hospital referral rate and high patient satisfaction are promising but, given the descriptive single-arm design, cannot by themselves establish appropriate triage, clinical safety, or an effect on emergency department use. These findings provide a sound rationale for a future comparative study designed to evaluate clinical safety and the impact of the model on emergency department utilisation and to inform its further development and geographical expansion.

## Figures and Tables

**Table 1:** Equipment used by Docadom physicians.

Category	Equipment
Vital sign monitoring	Pulse oximeter Automated sphygmomanometer Infrared thermometer
Cardiac assessment	Stethoscope Portable 12-lead electrocardiography with digital interface
Organ assessment	Smartphone-connected handheld ultrasound probe for cardiac, lung, pleural, abdominal, vascular scanning
Bedside tests	Quick tests for COVID-19, influenza A/B, respiratory syncytial virus, streptococcus; spot urine sample, pregnancy test Point-of-care testing device with measurement of C-reactive protein, N-terminal pro B-type natriuretic peptide, D-dimers (results available within 10 min)
Laboratory diagnostics	All blood tests (through a collaboration with a laboratory) with results within 2 h All types of biological cultures
Medications	Emergency kit with enough medication to provide 24 h of treatment (oral, topical, injectable)
Documentation	Laptop with electronic patient record Secure Swiss cloud storage
Communication and support	Encrypted smartphone with mobile application for patients, physician interface and dedicated web application, all developed by Docadom Back-office coordination with triage by a trained emergency nurse
Logistics	Electric cargo bike with lockable front storage compartment

**Table 2:** Patient demographics and clinical characteristics, overall and by age group.

	All Patients (*n* = 1736)	Age Group		
		<75 Years (*n* = 987)	≥75 Years (*n* = 749)	*p*-Value
Sex, *n* (%)				0.27
Female	1150 (66.2)	643 (65.1)	507 (67.7)	
Male	586 (33.8)	344 (34.9)	242 (32.3)	
Person requesting the consultation, *n* (%) consultation				<0.001
Patient	911 (52.5)	839 (85.0)	72 (9.6)	
Relative	77 (4.4)	44 (4.5)	33 (4.4)	
Nurse	694 (40.0)	88 (8.9)	606 (80.9)	
Primary care physician	54 (3.1)	16 (1.6)	38 (5.1)	
Mode of request, *n* (%)				<0.001
Mobile application	422 (24.3)	370 (37.5)	52 (6.9)	
Phone call	1314 (75.7)	617 (62.5)	697 (93.1)	
Times, min				
Waiting	93 [42, 170]	85 [32, 157]	105 [52, 184]	<0.001
Travel	14 [9.0, 21]	14 [8.0, 21]	14 [10, 21]	0.11
Consultation	35 [25, 48]	31 [22, 42]	42 [30, 54]	<0.001
Bedside tests				
Quick tests	383 (22.1)	249 (25.2)	134 (17.9)	<0.001
Point-of-care testing	311 (17.9)	139 (14.1)	172 (23.0)	<0.001
Electrocardiography	152 (8.8)	64 (6.5)	88 (11.7)	<0.001
Point-of-care ultrasound	140 (8.1)	64 (6.5)	76 (10.1)	0.006
Laboratory tests	186 (10.7)	102 (10.3)	84 (11.2)	0.56
Advanced procedures ^a^	107 (6.2)	60 (6.1)	47 (6.3)	0.11
Referral to a radiological centre ^b^	95 (5.5)	50 (5.1)	45 (6.0)	0.18
Outcome of the consultation				<0.001
Health issue solved	1393 (80.2)	867 (87.9)	526 (70.2)	
Follow-up by Docadom	109 (6.3)	34 (3.4)	75 (10.0)	
Follow-up by primary care physician	152 (8.8)	59 (6.0)	93 (12.5)	
Referral to hospital	82 (4.7)	27 (2.7)	55 (7.3)	

Values are numbers (%) or medians [interquartile ranges]. ^a^ Including intravenous infusion of medication, intravenous rehydration, wound stitches, abscess incision, and urinary bladder catheterisation. ^b^ Referral to a radiological centre was organised when a computed tomography scan, X-ray, ultrasound exam or magnetic resonance imaging was necessary.

**Table 3:** Diagnoses after completion of the consultation. Values are the number (%) of patients. The overall distribution of diagnoses differed significantly between the two age groups (*p* < 0.001, global chi-square comparison across all categories); this global *p*-value should not be interpreted as a significant difference for each individual category.

Number of Patients (%)	All Patients (*n* = 1736)	Age Group	
		<75 Years (*n* = 987)	≥75 Years (*n* = 749)
Ear, nose and throat infection, epistaxis and bronchitis	446 (25.7)	354 (35.9)	92 (12.3)
Soft tissue trauma	136 (7.8)	54 (5.5)	82 (10.9)
Acute cervical or lower back pain syndrome	97 (5.6)	70 (7.1)	27 (3.6)
Gastroenteritis and dehydration	86 (5.0)	48 (4.9)	38 (5.1)
Cancer and general decline in the elderly	80 (4.6)	23 (2.3)	57 (7.6)
Chronic obstructive pulmonary disease and asthma	66 (3.8)	31 (3.1)	35 (4.7)
Pneumonia	65 (3.7)	32 (3.2)	33 (4.4)
Functional abdominal pain	65 (3.7)	41 (4.2)	24 (3.2)
Arthritis (gout included)	60 (3.5)	27 (2.7)	33 (4.4)
Congestive heart failure	59 (3.4)	10 (1.0)	49 (6.5)
Migraines, headaches and vestibular neuronitis	58 (3.3)	38 (3.9)	20 (2.7)
Hypertension and hypotension	53 (3.1)	17 (1.7)	36 (4.8)
Cutaneous issue, zona	50 (2.9)	37 (3.7)	13 (1.7)
Lower urinary tract infection	46 (2.6)	23 (2.3)	23 (3.1)
Psychiatric disorder	44 (2.5)	26 (2.6)	18 (2.4)
Renal disease (e.g., upper urinary tract infection and lithiasis)	22 (1.3)	8 (0.8)	14 (1.9)
Arrhythmia (e.g., atrial fibrillation)	21 (1.2)	8 (0.8)	13 (1.7)
Ocular disease	20 (1.2)	14 (1.4)	6 (0.8)
Appendicitis, cholecystitis, diverticulitis and colitis	19 (1.1)	4 (0.4)	15 (2.0)
Acute coronary syndrome	14 (0.8)	7 (0.7)	7 (0.9)
Dermohypodermitis and abscess	12 (0.7)	2 (0.2)	10 (1.3)
Dementia and confusional state	12 (0.7)	1 (0.1)	11 (1.5)
Bone fracture and joint sprain	12 (0.7)	4 (0.4)	8 (1.1)
Head trauma	8 (0.5)	1 (0.1)	7 (0.9)
Allergy	8 (0.5)	7 (0.7)	1 (0.1)
Pulmonary embolism and deep venous thrombosis	3 (0.2)	1 (0.1)	2 (0.3)
Other	174 (10.0)	99 (10.0)	75 (10.0)

## Data Availability

The datasets used or analysed during the current study are available from the corresponding author on reasonable request.

## Supplementary Materials

The following supporting information can be downloaded at: https://www.mdpi.com/article/10.3390/jcm15156089/s1, File S1: STROBE Statement—checklist of items that should be included in reports of observational studies.

## Abbreviations

ECG	electrocardiogram
IQR	interquartile range
POCT	point-of-care testing
POCUS	point-of-care ultrasound
