# Supplementary material for: Descriptive Analysis of the First 12-Month Activity of Docadom, a Private Physician-Led Urgent In-Home Consultation Service
Source: J Clin Med. 2026 Aug 5;15(15):6089. doi: 10.3390/jcm15156089 (PMC13467185; doi:10.3390/jcm15156089)
Supplement: Supplementary file 1 [file jcm-15-06089-s001.zip › jcm-4434922-supplementary.pdf]

File S1: STROBE Statement—checklist of items that should be included in reports of observational studies

	Item No	Recommendation
**Title and abstract**	1	(*a*) Indicate the study's design with a commonly used term in the title or the abstract => DONE
		(*b*) Provide in the abstract an informative and balanced summary of what was done and what was found => DONE
**Introduction**		
Background/rationale	2	Explain the scientific background and rationale for the investigation being reported => DONE
Objectives	3	State specific objectives, including any prespecified hypotheses => DONE
**Methods**		
Study design	4	Present key elements of study design early in the paper => Methods §2.1 (retrospective descriptive analysis).
Setting	5	Describe the setting, locations, and relevant dates, including periods of recruitment, exposure, follow-up, and data collection => Methods §2.1
Participants	6	(*a*) *Cohort study*—Give the eligibility criteria, and the sources and methods of selection of participants. Describe methods of follow-up<br>*Case-control study*—Give the eligibility criteria, and the sources and methods of case ascertainment and control selection. Give the rationale for the choice of cases and controls<br>*Cross-sectional study*—Give the eligibility criteria, and the sources and methods of selection of participants
		(*b*) *Cohort study*—For matched studies, give matching criteria and number of exposed and unexposed<br>*Case-control study*—For matched studies, give matching criteria and the number of controls per case<br>*=>Methods §2.1 (all patients treated in the first 12 months), §2.2 (adults >16 years; request pathways).*
Variables	7	Clearly define all outcomes, exposures, predictors, potential confounders, and effect modifiers. Give diagnostic criteria, if applicable => Methods §2.3 (full list of variables and outcomes); diagnostic categories in Table 3.
Data sources/ measurement	8*	For each variable of interest, give sources of data and details of methods of assessment (measurement). Describe comparability of assessment methods if there is more than one group => Methods §2.2 and §2.3 (recorded variables).
Bias	9	Describe any efforts to address potential sources of bias => Discussion (limitations)
Study size	10	Explain how the study size was arrived at => Methods §2.1 — no a priori sample size; all patients seen during the 12-month period were included (n = 1736; Results).
Quantitative variables	11	Explain how quantitative variables were handled in the analyses. If applicable, describe which groupings were chosen and why => Methods §2.4
Statistical methods	12	(*a*) Describe all statistical methods, including those used to control for confounding
		(*b*) Describe any methods used to examine subgroups and interactions
		(*c*) Explain how missing data were addressed
		(*d*) *Cohort study*—If applicable, explain how loss to follow-up was addressed<br>*Case-control study*—If applicable, explain how matching of cases and controls was addressed

		Cross-sectional study—If applicable, describe analytical methods taking account of sampling strategy
		(*e*) Describe any sensitivity analyses<br>=> Methods §2.4
**Results**		
Participants	13*	(a) Report numbers of individuals at each stage of study—eg numbers potentially eligible, examined for eligibility, confirmed eligible, included in the study, completing follow-up, and analysed
		(b) Give reasons for non-participation at each stage
		(c) Consider use of a flow diagram<br>=> (a) Results (1,736 consultations); Table 2. (b) Not applicable (all consecutive patients included). (c) Patient workflow shown in Figure 2 (no CONSORT-type flow required, as all patients were included).
Descriptive data	14*	(a) Give characteristics of study participants (eg demographic, clinical, social) and information on exposures and potential confounders
		(b) Indicate number of participants with missing data for each variable of interest
		(c) *Cohort study*—Summarise follow-up time (eg, average and total amount)<br>=> Results tables 2 and 3
Outcome data	15*	*Cohort study*—Report numbers of outcome events or summary measures over time
		Case-control study—Report numbers in each exposure category, or summary measures of exposure
		Cross-sectional study—Report numbers of outcome events or summary measures<br>=> Results tables 2 and
Main results	16	(*a*) Give unadjusted estimates and, if applicable, confounder-adjusted estimates and their precision (eg, 95% confidence interval). Make clear which confounders were adjusted for and why they were included
		(*b*) Report category boundaries when continuous variables were categorized
		(*c*) If relevant, consider translating estimates of relative risk into absolute risk for a meaningful time period<br>=> Results tables 2 and
Other analyses	17	Report other analyses done—eg analyses of subgroups and interactions, and sensitivity analyses<br>=> Subgroup analyses by age group reported throughout Results and in Tables 2 and 3; no additional analyses performed.
**Discussion**		
Key results	18	Summarise key results with reference to study objectives => Discussion, opening paragraphs; Abstract (Conclusions)
Limitations	19	Discuss limitations of the study, taking into account sources of potential bias or imprecision. Discuss both direction and magnitude of any potential bias => Discussion (limitations)
Interpretation	20	Give a cautious overall interpretation of results considering objectives, limitations, multiplicity of analyses, results from similar studies, and other relevant evidence => Discussion; Conclusion (interpretation focused on feasibility and acceptability; comparison with hospital-at-home and diversion literature).
Generalisability	21	Discuss the generalisability (external validity) of the study results => Discussion (Limitations): findings may not be generalisable to rural areas or other health systems without adaptation.
**Other information**		

Funding	22	Give the source of funding and the role of the funders for the present study and, if applicable, for the original study on which the present article is based => Funding statement (no external funding); Conflicts of Interest statement.

*Give information separately for cases and controls in case-control studies and, if applicable, for exposed and unexposed groups in cohort and cross-sectional studies.

Note: An Explanation and Elaboration article discusses each checklist item and gives methodological background and published examples of transparent reporting. The STROBE checklist is best used in conjunction with this article (freely available on the Web sites of PLoS Medicine at http://www.plosmedicine.org/, Annals of Internal Medicine at http://www.annals.org/, and Epidemiology at http://www.epidem.com/). Information on the STROBE Initiative is available at www.strobe-statement.org.
